# Announcements

**Published:** 1855-09

**Authors:** 


*Announcements.*—From every quarter the college circulars have been pouring in. A neatly executed favor of the kind came to our table from our neighbors of "St. Louis University" which, in a very inviting manner, calls the attention of the profession to their present prosperous condition, and their abundant preparation to meet the wants and necessities for the future; and then just upon the heels of this again, our northern neighbors of the "Rush School" show their readiness, fitness, and competency for teaching, which we do not doubt for one moment, and from the manner and spirit of their "*pronunciamento*" one would infer that they expected a "rush" into their halls the coming session, in which expectations we hope they will be amply gratified. Then comes the unpretending "declaration of principles" from the Jefferson and one from each of the other institutions from that "bright focus" of medical literature, Philadelphia, and these again are followed by swarms of visitors in their various dresses, "blue spirits and white, red colored and yellow," (pardon us for this change,) and from every quarter "mingling" around us, displaying each its own typographical superiority, and yet in no one have we yet found a sentence which would convey the idea that the particular school whence it emanated stood pre-eminently above its neighbors, or that the "body medical" would suffer a serious abatement should they not have existed. They all seem to be disposed to pursue the even tenor of their way, ready to do good service, decrying none nor indulging in untried transcendental ideas of self superiority.

For all such we entertain the kindest feelings, because they all contribute largely to American Medical Literature, and we ardently wish for the success of each. We have sent out our "*messenger*," and trust it will be received in the same spirit of kindness in which it is sent on its "greeting errand." It gives notice that we "still live and have our being," and are inspired by the hope "that is within us," that we will continue to do so. From present indications our Class will be as large, if not larger, than last session, and we can assure our medical friends that we are fully ready and prepared for the discharge of our duties, and that whatever of State pride is entertained as growing out of the existence of the Medical Department of the University, it shall not be disappointed so long as they, (as all good citizens should,) feel an interest in the welfare of their own State literary and scientific enterprises. The best and most liberal minded men of other States, have ever exhibited the deepest interest in their own institutions, and will point the stranger with exultation to the literary and scientific enterprises towering up in their midst. To a young State like ours, in whose valleys the echo of the red-savage is still faintly heard, it is a source of pride, as well as promise, that the means of instruction in literature and science are so abundant and within the reach of every one desirous of prosecuting them. Free instruction is yet to constitute a feature of our systems, having in view the march of mind and accords with the spirit of our institutions, and of progress. We will never come up to the letter of republicanism until our State governments provide most liberally to this end. This feature, in part. has been adopted in the Medical department of the State University, but the time is coming when the people will provide liberally for all branches of instruction, and open the doors wide for the admission of all.

Our Session will open on the first of November next, at which time we hope as many Students will be present as possible. Prof. Allen will deliver the Introductory, which will doubtless prove a treat to all who can arrive in time to hear him. After the introductory, each Professor will, in his place in the Halls of the College, deliver the introductory to his Course, which will also be important, because containing a synopsis of the subjects to be treated by him, and their arrangement.

